# Acute Endurance Exercise Induces Nuclear p53 Abundance in Human Skeletal Muscle

**DOI:** 10.3389/fphys.2016.00144

**Published:** 2016-04-26

**Authors:** Bill Tachtsis, William J. Smiles, Steven C. Lane, John A. Hawley, Donny M. Camera

**Affiliations:** ^1^Centre for Exercise and Nutrition, Mary MacKillop Institute for Health Research, Australian Catholic UniversityMelbourne, VIC, Australia; ^2^Research Institute for Sport and Exercise Sciences, Liverpool John Moores UniversityLiverpool, UK; ^3^Exercise and Nutrition Research Group, School of Medical Sciences, RMIT UniversityMelbourne, VIC, Australia

**Keywords:** mitochondrial biogenesis, autophagy, mitochondrial turnover, cell signaling, skeletal muscle

## Abstract

**Purpose:** The tumor suppressor protein p53 may have regulatory roles in exercise response-adaptation processes such as mitochondrial biogenesis and autophagy, although its cellular location largely governs its biological role. We investigated the subcellular localization of p53 and selected signaling targets in human skeletal muscle following a single bout of endurance exercise.

**Methods:** Sixteen, untrained individuals were pair-matched for aerobic capacity (VO_2peak_) and allocated to either an exercise (EX, *n* = 8) or control (CON, *n* = 8) group. After a resting muscle biopsy, EX performed 60 min continuous cycling at ~70% of VO_2peak_ during which time CON subjects rested. A further biopsy was obtained from both groups 3 h post-exercise (EX) or 4 h after the first biopsy (CON).

**Results:** Nuclear p53 increased after 3 h recovery with EX only (~48%, *p* < 0.05) but was unchanged in the mitochondrial or cytoplasmic fractions in either group. Autophagy protein 5 (Atg-5) decreased in the mitochondrial protein fraction 3 h post-EX (~69%, *P* < 0.05) but remained unchanged in CON. There was an increase in cytoplasmic levels of the mitophagy marker PINK1 following 3 h of rest in CON only (~23%, *P* < 0.05). There were no changes in mitochondrial, nuclear, or cytoplasmic levels of PGC-1α post-exercise in either group.

**Conclusions:** The selective increase in nuclear p53 abundance following endurance exercise suggests a potential pro-autophagy response to remove damaged proteins and organelles prior to initiating mitochondrial biogenesis and remodeling responses in untrained individuals.

## Introduction

Exercise represents a major challenge to whole-body homeostasis, and in an attempt to meet this challenge, a myriad of acute and adaptive responses take place at the cellular and systemic levels that function to minimize these widespread disruptions (Hawley et al., 2014). In this regard, human skeletal muscle displays remarkable plasticity, with the capacity to alter both the type and amount of protein in response to disruptions in cellular homeostasis induced by the habitual level of contractile activity, the prevailing substrate availability, and environmental conditions (Hawley and Zierath, 2004; Hawley et al., 2011).

Mitochondrial biogenesis is the primary skeletal muscle adaptation that occurs in response to endurance exercise training. Mitochondrial biogenesis requires the coordinated transcription and eventual synthesis of several nuclear- and mitochondrial- DNA (mtDNA)-encoded proteins that are either incorporated into existing mitochondria or contribute to the formation of new organelles (Yan et al., 2012). Anabolic processes such as the synthesis of new contractile proteins and mitochondrial biogenesis have been widely studied *in vivo*. In contrast, less is known about the catabolic processes that contribute to exercise-induced adaptation.

Autophagy is a catabolic cellular process responsible for the degradation of cellular constituents such as soluble proteins, damaged organelles (e.g., mitochondria) and intracellular pathogens (Mizushima and Klionsky, 2007). Autophagy involves the sequestration of cellular constituents into double-membrane vesicles called autophagosomes, which deliver their “cargo” to the lysosomes for degradation. We (Smiles et al., 2015) and others (Fry et al., 2013; Schwalm et al., 2015) have shown that exercise modulates the expression of select proteins involved in autophagy in human skeletal muscle.

The tumor suppressor p53 is well-known for its ability to repress erroneous DNA replication via cell cycle arrest, differentiation, senescence, quiescence, and/or apoptosis (Kruiswijk et al., 2015). A growing body of evidence also implicates p53 in exercise adaptation responses due to its regulation of protein targets regulating oxidative metabolism, autophagy and mitochondrial biogenesis processes (Bartlett et al., 2014). Of note, the subcellular localization of p53 has been shown to modulate its function. For example, cytoplasmic p53 is inhibitory toward autophagy, whereas nuclear p53 promotes autophagy by transactivating several genes modulating the energy-sensitive mammalian target of rapamycin (mTOR)/AMP-activated protein kinase (AMPK) pathway (Maiuri et al., 2010). Studies in rodent skeletal muscle have shown that acute exercise induces the translocation of p53 from the nucleus to the mitochondria where it interacts with the mitochondrial transcription factor A (Tfam) to positively affect mtDNA transcription (Saleem and Hood, 2013). However, few investigations have quantified mitochondrial protein abundance in human skeletal muscle or determined the effects of exercise on p53 expression in the different cellular protein pools. Accordingly, the aim of the current investigation was to determine the abundance of p53 and its signaling targets in the nuclear, cytoplasmic, and mitochondrial protein fractions following an acute bout of endurance exercise. We hypothesized aerobic-based exercise would induce the translocation of p53 to the mitochondria and subsequently prioritize the expression of protein targets regulating mitochondrial biogenesis over autophagy processes.

## Methods

### Subjects

Sixteen healthy, untrained, male subjects [age 21.3 ± 4.0 years, body mass (BM) 78.7 ± 8.6 kg, peak oxygen uptake (VO_2peak_) 40.4 ± 6.5 mL·kg^−1^·min^−1^, peak power output (PPO) 2.9 ± 0.4 W/kg] were recruited for this study. Subjects were provided with oral and written information about the purpose, nature, and potential risks involved with the study, and written informed consent was obtained prior to participation. The study was approved by the RMIT University Human Research Ethics Committee and conducted in conformity with the policy statement regarding the use of human subjects according to the latest revision of the Declaration of Helsinki.

### Experimental design

The study employed a between-subjects design where subjects were pair-matched for aerobic capacity (VO_2peak_) and allocated to either an exercise (*n* = 8) or control (*n* = 8) group for the experimental trial. Due to insufficient tissue sample size, two participants from the control group were excluded from data analysis (*n* = 6).

### Preliminary testing

#### VO_2peak_

VO_2peak_ was determined during an incremental test to volitional fatigue on a Lode cycle ergometer (Groningen, The Netherlands) as previously described (22). In brief, subjects commenced cycling at a workload equivalent to 2 W·kg body mass (BM) for 150 s. Thereafter, the workload was increased by 25 W every 150 s until volitional fatigue, which was defined as the inability to maintain a pedaling cadence >70 rev.min^−1^. VO_2peak_ was determined 2 weeks prior to experimental trials during which time subjects maintained their habitual diet and physical activity patterns.

### Diet/exercise control

Before experimental trials (described subsequently), subjects were instructed to refrain from exercise training and vigorous physical activity and alcohol and caffeine consumption for a minimum of 48 h. Subjects were provided with standardized pre-packed meals that consisted of 3 g carbohydrate/kg BM, 0.5 g protein/kg BM, and 0.3 g fat/kg BM consumed as the final caloric intake the evening before reporting for an experimental trial.

### Experimental trials

On the morning of the experimental trial, subjects reported to the laboratory after a ~10-h overnight fast. After resting in the supine position for ~15 min and under local anesthesia (2–3 mL of 1% Xylocaine), a resting biopsy was obtained from the *vastus lateralis* from all participants using a 5-mm Bergstrom needle modified with suction. Subjects in EX then completed the exercise intervention which consisted of 60 min cycling at a power output corresponding to ~70% of their VO_2peak_. This exercise bout was chosen as it has been previously shown to elicit a substantial metabolic perturbation (Camera et al., 2010). During this time, subjects in CON rested. Three hours following the completion of the exercise bout or 4 h after the first resting biopsy, another muscle biopsy was obtained from EX and CON groups, respectively. Each muscle biopsy was taken from a separate site 2–3 cm distal from each other and all samples were dissected free from blood and connective tissue and snap frozen in liquid nitrogen before being stored at −80°C until further analyses.

### Mitochondrial fractionation

Muscle samples (~50–70 mg) were homogenized by hand using an ice-cold glass Dounce homogenizer in a solution containing 800 μL BSA/ Mitochondrial Isolation Reagent A supplemented with protease inhibitors (Thermo Fisher, Melbourne Australia, Cat No. 1859692). Muscle homogenates were then transferred into an ice-cold 2 mL Eppendorf tube and centrifuged (700 g; 15 min; 4°C). The resulting supernatant was transferred into a new 1.5 mL Eppendorf tube and subsequently centrifuged (10,500 g; 15 min; 4°C). The supernatant was removed and the remaining pellet was washed with 500 μL of Mitochondrial Reagent C (Thermo Fisher, Melbourne Australia, Cat No., #1859694) and centrifuged (10,500 g; 15 min; 4°C). The supernatant was removed and 100 μL of 2% CHAPS solution (Sigma-Aldrich, Castle Hill, Australia, Cat No.:C3023) was added to the pellet and mixed by vortexing for 1 min to perturb the pellet before being centrifuged at high-speed for 2 min. The supernatant (mitochondrial protein pool) was removed and placed on ice for subsequent determination of protein concentration using a bicinchoninic acid (BCA) protein assay (Pierce, Rockford, USA).

### Nuclear and cytoplasmic fractionation

Nuclear and cytoplasmic fractions were prepared using a commercially available nuclear extraction kit (Pierce, Rockford, USA, Cat No.: 78833). Approximately 20 mg of skeletal muscle was homogenized in ice-cold Cytoplasmic Extraction Reagent (CER I) buffer containing a protease inhibitor cocktail. Homogenates were centrifuged at 16,000 g for 10 min at 4°C, and the supernatant (cytoplasmic fraction) was removed and placed on ice. Following a series of washes, nuclear proteins were extracted in high salt Nuclear Extraction Reagent (NER) buffer supplemented with protease inhibitors. Following 40 min of incubation, samples were re-centrifuged at 16,000 g, and the supernatant (nuclear fraction) was placed on ice. Small amounts of both the nuclear and cytoplasmic supernatant were subsequently used for determination of protein concentration using a BCA protein assay (Pierce, Rockford, USA). Due to limited tissue availability for some time points, nuclear, and cytoplasmic fractionation analysis was only performed on *n* = 5–6. Analysis of nuclear PGC-1α expression was only conducted on four subjects due to a failed gel to membrane transfer and poor chemiluminescence signal on exposure.

### Immunoblotting

Mitochondrial, nuclear, and cytoplasmic lysates were re-suspended in Laemelli sample buffer, separated using 4–20% Stain-Free Precast gels (Bio-Rad, Richmond, USA) and transferred to polyvinylidinefluoride (PVDF) membranes, blocked with 5% non-fat milk, washed with 10 mM Tris·HCl, 100 mM NaCl, and 0.02% Tween 20 and incubated with a primary antibody (1:1000, unless stated otherwise) overnight at 4°C. Membranes were incubated the next day with a secondary antibody (1:2000) and proteins were detected via enhanced chemiluminescence (Pierce Biotechnology, Rockford, USA) and quantified by densitometry (Chemidoc, BioRad, Gladesville, Australia). Ten microgram of protein was loaded into each well for all mitochondrial fractions, while 13 and 25 μg were loaded for nuclear and cytoplasmic fractions, respectively. Antibodies directed against p53 (1:500), Tfam (Clone Number D5C8), Mitofusin-2 (Clone Number D1E9), apoptosis inducing factor (AIF) (Clone Number D39D2), AMPKα, autophagy-related gene protein 5 (Atg5), unc-5-like kinase 1 (ULK1; Clone Number D9D7), p62, PTEN-induced putative protein kinase 1 (PINK1; Clone Number D8G3), Parkin, and dynamin-related protein 1 (DRP1; Clone Number D6C7) were purchased from Cell Signaling Technology (Danvers, USA). Microtubule-associated protein one light chain 3b (LC3b) and *PPAR*γ-coactivator-1α (PGC-1α; N-terminal) antibodies were purchased from Abcam (Cambridge, UK). COXIV (Clone Number 3E11), histone 2B (H2B) (Clone Number V119), and GAPDH (Clone Number 14C10) were purchased from Cell Signaling Technology and were used to assess the purity of the mitochondrial, nuclear, and cytoplasmic fractions, respectively. All data for each fraction were normalized to the total protein loaded (Supplementary Figure 1) into each lane using Stain-free technology (Gurtler et al., 2013) as performed previously (Smiles et al., 2015).

### Statistical analysis

Statistical analysis was conducted using Sigma Plot (Version 12.5). Data were analyzed by a two-way analysis of variance (ANOVA) with time and treatment as factors to compare differences between treatments over time. Where there were significant main effects for treatment or time, pre-planned Student-Newman-Kuels post hoc tests were used to locate the differences within each group separately. When tests for normality and/or equal variance failed, data were log-transformed. Specifically, Atg-5 and Mitofusin-2 were log-transformed. All data in text and figures are presented as mean ± SD with *P* < 0.05 indicating statistical significance.

## Results

### Purity of cellular fractions

**Figure 1:**
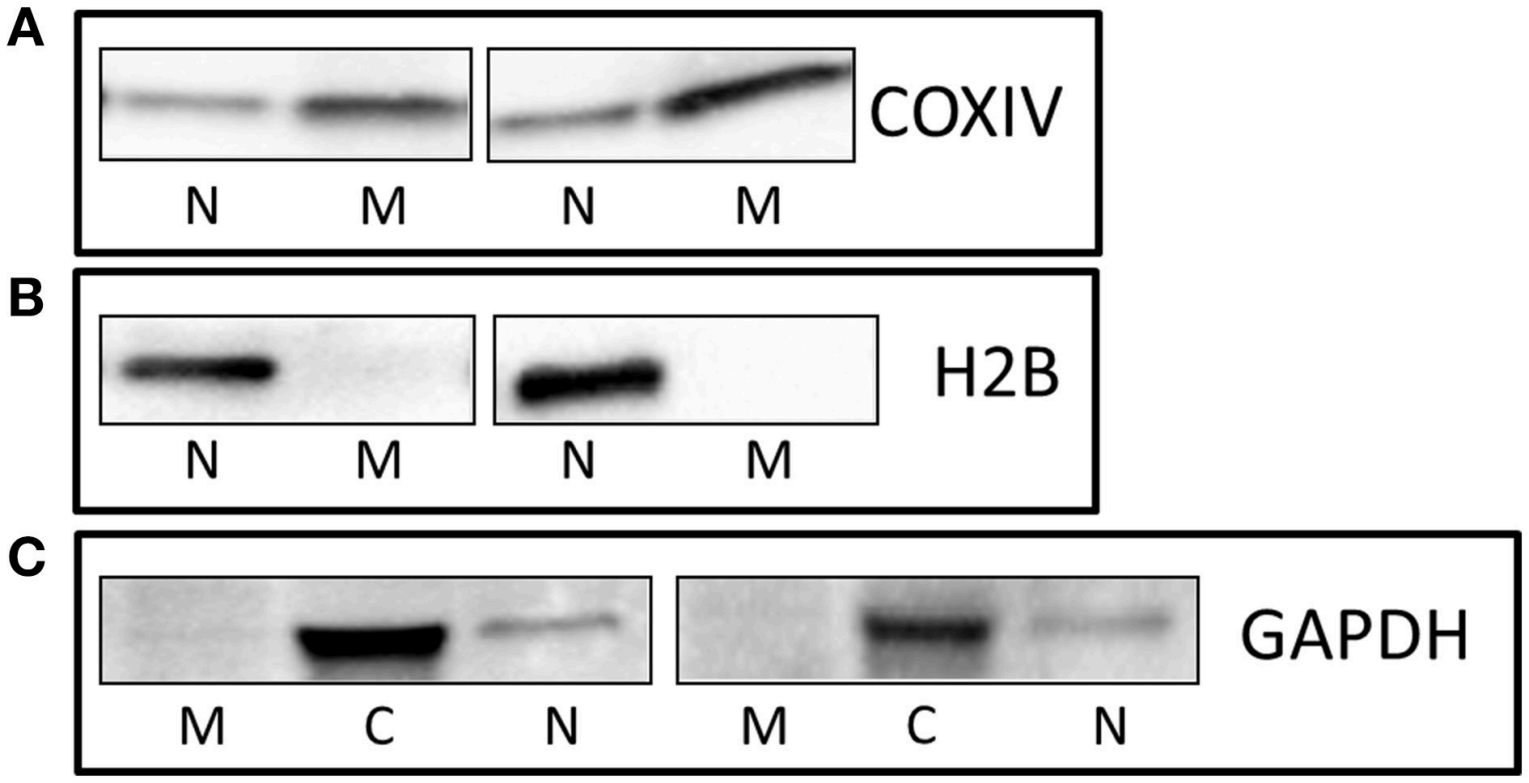
**Enrichment and purity of protein fractions**. Representative Western Blots showing that **(A)** COXIV is highly abundant in the mitochondrial fraction **(A)**, and H2B **(B)**, and GAPDH **(C)** were enriched in nuclear and cytoplasmic fractions, respectively. M, Mitochondria; N, Nuclear; C, Cytoplasmic.

Equal amounts of protein from the mitochondrial (M), cytoplasmic (C), and nuclear (N) fractions were analyzed using standard Western Blotting. The mitochondrial fraction was highly abundant for COX-IV (Figure 1A), while the nuclear and cytoplasmic protein pools were also highly pure for histone 2B (H2B; Figure 1B) and the glycolytic enzyme GAPDH (Figure 1C), respectively.

### Subcellular localization of p53 and PGC-1α abundance

**Figure 2:**
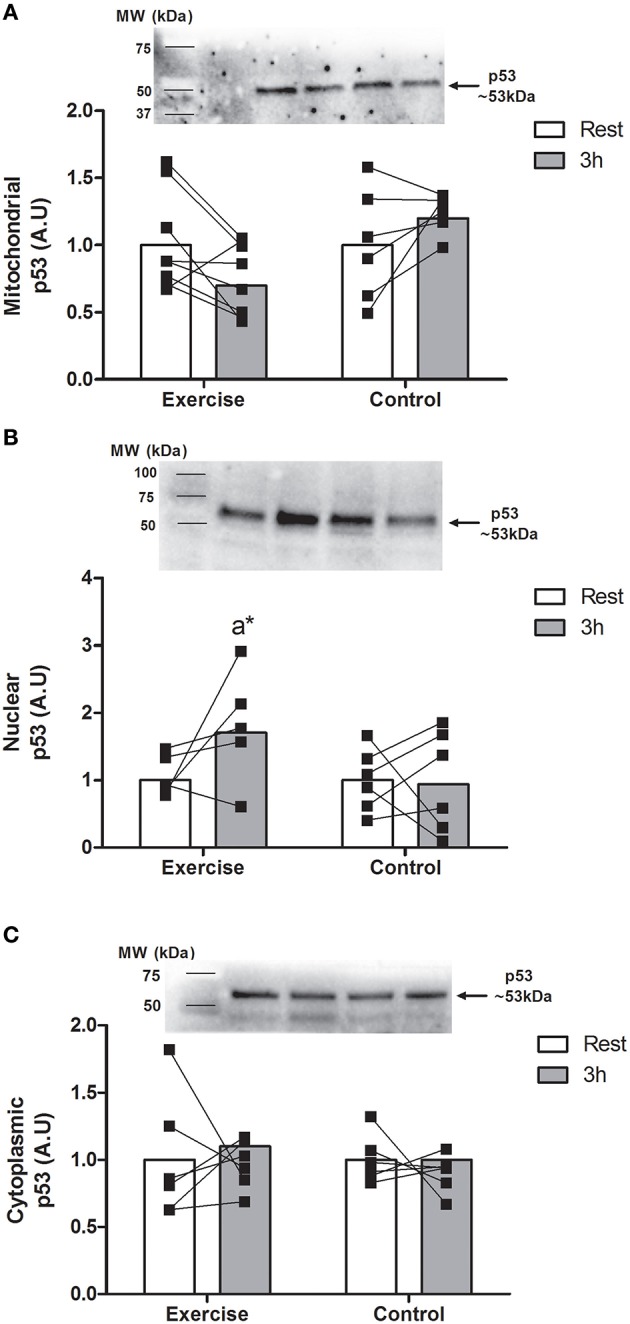
**Changes in mitochondrial (A; *n* = 8 EX; *n* = 6 CON), nuclear (B; *n* = 5 EX; *n* = 6 CON), and cytoplasmic (C; *n* = 6 EX; *n* = 6 CON) expression of p53 at rest and 3 h following endurance exercise (60 min cycling ~70% VO_2peak_) or 4 h rest (CON)**. All values are expressed relative to stain free gel and presented in arbitrary (means ± *SD*) with statistical significance established when *P* < 0.05. Different vs. ^a^rest within condition; *different than 3 h post CON.

**Figure 3:**
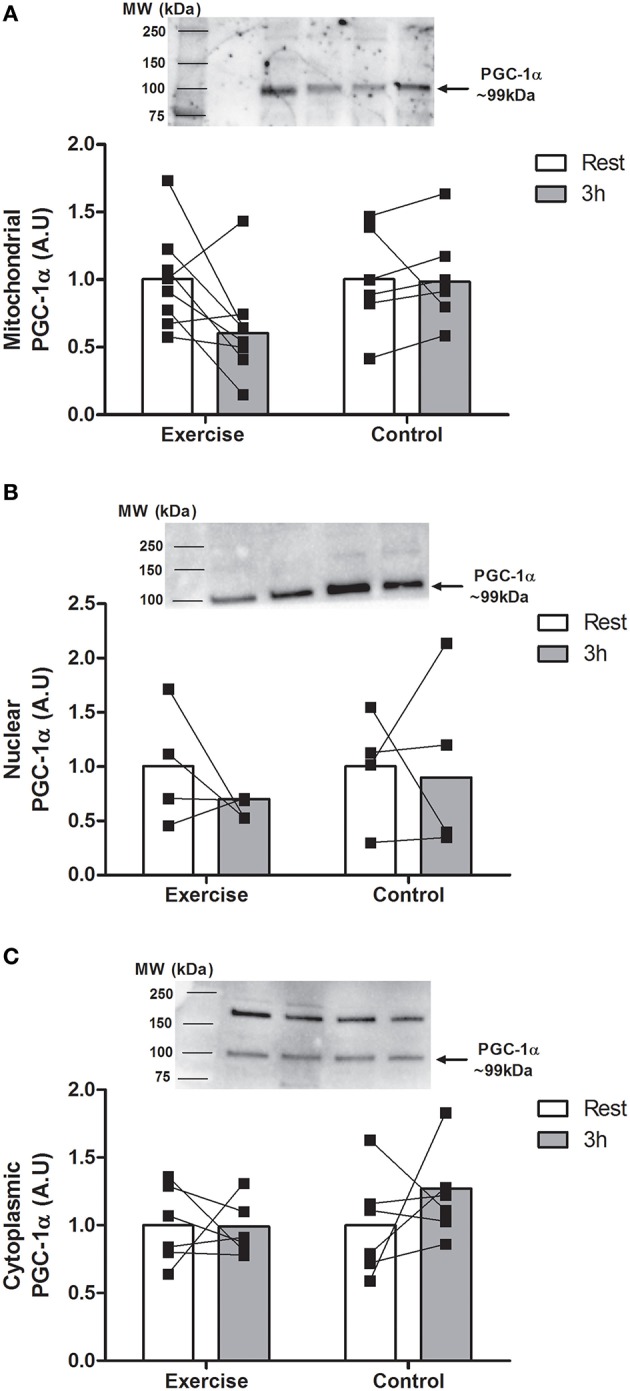
**Expression of PGC-1α in the mitochondria (A; *n* = 8 EX; *n* = 6 CON), nucleus (B; *n* = 4 EX, *n* = 4 CON), and cytoplasm (C; *n* = 6 EX; *n* = 6 CON) at rest and 3 h following endurance exercise (60 min cycling ~70% VO_2peak_) or 4 h rest (CON)**. All values are expressed relative to stain free gel and presented in arbitrary (means ± *SD, n* = 8) with statistical significance established when *P* < 0.05.

There was a main effect for time with nuclear p53 expression only (*P* < 0.05). There was an increase in nuclear p53 abundance after 3 h in EX only (~48%, *P* < 0.05; Figure 2). In contrast, there were no changes in cytoplasmic or mitochondrial p53 expression in either group. PGC-1α protein abundance remained unchanged in all protein fractions for both groups (Figure 3).

### Coxiv, Mitofusin-2, Tfam, AIF, Atg5, and ULK-1 mitochondrial expression

**Figure 4:**
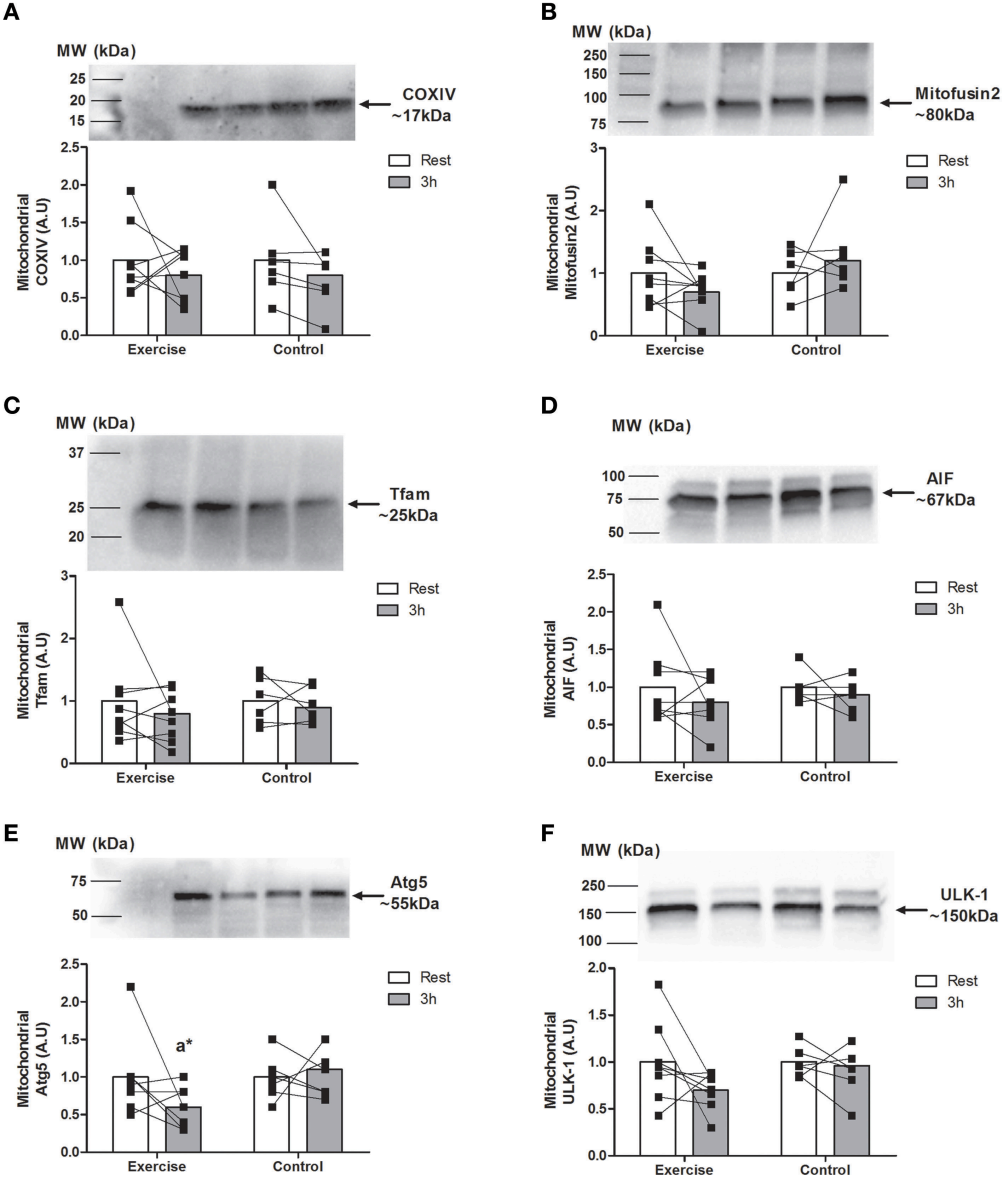
**Expression of proteins associated with mitochondrial remodeling at rest and 3 h following endurance exercise (60 min cycling ~70% VO_2peak_)**. Mitochondrial COXIV **(A)**, Mitofusin-2 **(B)**, Tfam **(C)**, AIF **(D)** Atg5 **(E)** and ULK-1 **(F)**. All values are expressed relative to stain free gel and presented in arbitrary (means ± *SD, n* = 8 EX; *n* = 6 CON) with statistical significance established when *P* < 0.05. Different vs. ^a^rest within condition; *different than 3 h post CON.

There were no changes between groups in the mitochondrial abundance of protein markers regulating mitochondrial function and fusion including COXIV (Figure 4A), Mitofusin-2 (Figure 4B), and Tfam (Figure 4C). Similarly, there were no differences in mitochondrial AIF (Figure 4D), or ULK-1 (Figure 4F) at any time. Total Atg-5 decreased in the mitochondrial fraction 3 h post-exercise with EX (~69%, *P* < 0.05) but not CON (Figure 4E).

### Autophagy-related protein cytoplasmic expression

**Figure 5:**
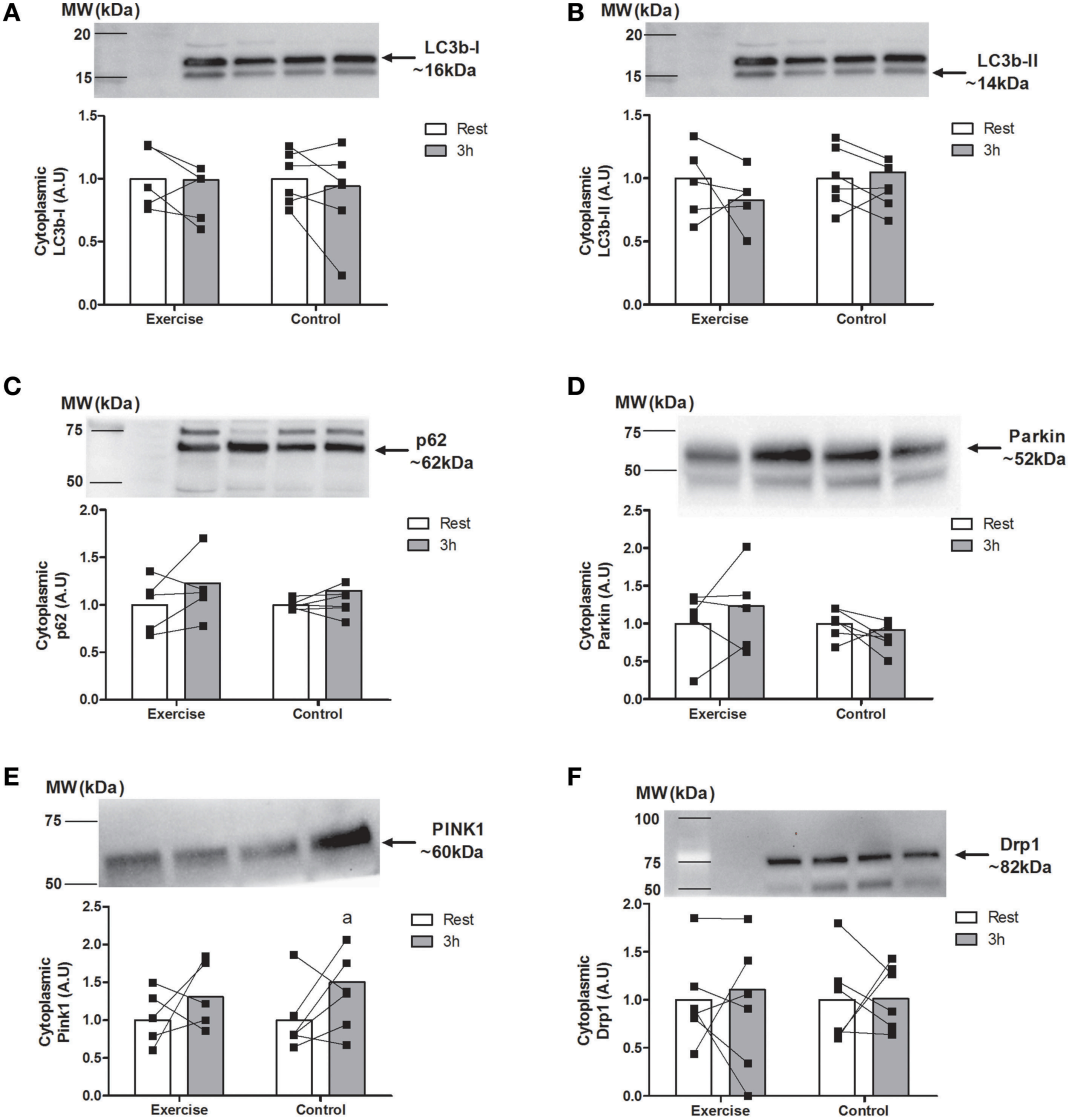
**Cytoplasmic and mitochondrial levels of markers of basal autophagy, autophagy, and mitophagy proteins at rest and 3 h following endurance exercise (60 min cycling ~70% VO_2peak_) LC3b-I (A; *n* = 5 EX; *n* = 6 CON), LC3b-II (B; *n* = 5 EX; *n* = 6 CON), p62 (C; *n* = 5 EX; *n* = 6 CON), Parkin (D; *n* = 5 EX; *n* = 6 CON), PINK1 (E; *n* = 5 EX; *n* = 6 CON), and DRP1 (F; *n* = 6 EX; *n* = 6 CON)**. All values are expressed relative to stain free gel and presented in arbitrary (means ± *SD*) with statistical significance established when *P* < 0.05. Different vs. ^a^rest within condition.

Cytoplasmic levels of LC3b-I, the lipid-conjugated LC3b-II and the substrate trafficking protein p62 did not change at any time point (Figures 5A–C). Similarly, there were no changes in the LC3b-II/I ratio following exercise (data no shown). There was a main effect for time for the mitophagy marker Pink1 (*P* < 0.05). Specifically, PINK1 expression increased at 3 h in the CON group only (~23%, *P* < 0.05) with no accompanying differences in its target Parkin (Figures 5D,E). No changes were detected for the outer mitochondrial membrane fission protein DRP1 (Figure 5F).

## Discussion

The apoptogenic protein p53 has recently emerged as a potential regulator of exercise-induced mitochondrial biogenesis, autophagy, and substrate metabolism responses in skeletal muscle (Saleem et al., 2009). While increased p53 phosphorylation has been reported from whole-muscle homogenates in humans (Bartlett et al., 2012, 2013; Camera et al., 2015), the regulation of p53 subcellular localization following exercise in human skeletal muscle has not been determined. We demonstrate for the first time an increase in nuclear p53 content following endurance exercise, although p53 mitochondrial abundance remained unchanged. We also observed a decrease in mitochondrial Atg5 post-exercise while there was a concomitant increase in cytoplasmic PINK1 in the non-exercise (control) group. This is the first human study to report subcellular changes in p53 and select markers of autophagy that ultimately contribute to the molecular basis promoting exercise adaptation responses.

Mitochondrial biogenesis is the hallmark intracellular adaptation to endurance exercise (Drake et al., 2016) and requires a finely-tuned transcription/translation of proteins encoded within nuclear and mitochondrial DNA (mtDNA) (Perez-Schindler and Philp, 2015). p53 has been recognized as a putative regulator of mitochondrial biogenesis due to seminal observations of attenuated steady-state mitochondrial content and markedly reduced exercise capacity in p53 knockout mice (Matoba et al., 2006; Park et al., 2009; Saleem et al., 2009). To elucidate a potential mechanism of action of p53-induced mitochondrial biogenesis, Saleem and Hood (2013) demonstrated that endurance exercise in rodents promoted p53 nuclear export and mitochondrial uptake where it formed a complex with Tfam at the mtDNA D-loop to upregulate mtDNA transcription. In contrast to our original hypothesis, we report an increase in nuclear p53 content concomitant with unchanged mitochondrial p53 and Tfam abundance following an acute bout of endurance exercise. While we cannot preclude possible differences in p53 activity (i.e., changes in phosphorylation and/or acetylation status), these results suggest that p53 does not accumulate in the mitochondria as a result of its nuclear expulsion in the acute recovery period (3 h) following endurance exercise in untrained human skeletal muscle.

No other study has investigated mitochondrial abundance of p53 in human skeletal muscle most likely because of the large amounts of tissue required (i.e., : >50 mg) and the small pool of proteins that compose this fraction (~10%). In this regard, we did observe a small “nuclear contamination” with our isolated mitochondria protein pool (Figure 1). While we cannot completely discount the presence of this nuclear protein impacting on the results obtained for our mitochondrial protein analysis, other studies in human skeletal muscle extracting nuclear and cytoplasmic fractions also show slight impurities in their isolated fractions (Little et al., 2010, 2011). Studies investigating p53 signaling responses following exercise in human skeletal muscle have primarily focused on the phosphorylation status at the Serine 15 residue as this post-translational modification is associated with enhanced stability and activity of the protein (Bartlett et al., 2014). Moderate-intensity continuous and high-intensity interval running have been shown to increase p53^Ser15^ phosphorylation in human skeletal muscle (Bartlett et al., 2012). Moreover, we (Camera et al., 2015) and others (Bartlett et al., 2013) have reported increased p53 phosphorylation when commencing exercise with low muscle glycogen concentration, suggesting a possible glycogen-mediated effect on p53 signaling. However, a confounding limitation of these studies is that they do not delineate the subcellular location of this p53 response. This information is essential as the biological function of p53 is dependent on its subcellular localization. For example, cytoplasmic p53 has been shown to inhibit autophagy, whereas nuclear p53 can promote autophagy (Maiuri et al., 2010).

A novel finding of the current study was the increase in nuclear p53 abundance post-exercise, indicating a p53-transcriptional stress response, of which could include autophagy (Feng et al., 2007). Autophagy involves formation of autophagosomes, vesicles that sequester and deliver cellular constituents to lysosomes for degradation in a process regulated by autophagy-related gene (Atg) proteins (Mizushima et al., 2011). Autophagy is stimulated by endurance exercise (Jamart et al., 2012a; Lira et al., 2013; Schwalm et al., 2015) due to its sensitivity to perturbations in cellular energy balance and mitochondrial respiration (Singh and Cuervo, 2011; Qiao et al., 2015). In the current study, there were no changes in ULK1 subcellular distribution, a proximal signaling Atg whose activity is regulated by AMPK (Egan et al., 2011; Kim et al., 2011). As we have previously shown that peak AMPK phosphorylation occurs within the first 30 min following-exercise (Camera et al., 2010), any AMPK-mediated changes in ULK1 activity may have occurred prior to our 3 h biopsy time point.

In contrast, mitochondrial levels of Atg5, an ubiquitin-like enzyme that contributes to expansion of the autophagosomal membrane (Mizushima et al., 2011), decreased with exercise. This finding suggests mitophagy (mitochondria-specific autophagy) may have been repressed following endurance exercise, although there were no accompanying changes in the cytoplasmic (mitochondria-containing) abundance of PINK1 and Parkin, proteins that specifically segregate damaged mitochondria for their preferential degradation by mitophagy (Narendra et al., 2010; Vives-Bauza et al., 2010). In addition, LC3b lipidation was unchanged post-exercise, increases of which, alongside a reduction in the “bridging” protein p62 that delivers substrates to autophagosomes (and undergoes degradation itself), can be used to infer autophagosome biogenesis (Tanida et al., 2008). There were also no differences in cytoplasmic DRP1, an outer mitochondrial membrane fission protein whose membrane scission assists mitophagic processes (Twig et al., 2008). Collectively, our results suggest that endurance exercise did not upregulate autophagic flux and may have temporarily attenuated mitophagy. Another consideration is that the reduced targeting of Atg5 to mitochondria following exercise was the result of preferential upregulation of the ubiquitin-proteasomal pathway (UPP). Indeed, gene expression of key ubiquitin ligases MuRF1 and Atrogin-1 peaks from 2–4 h following a single bout of endurance exercise (Louis et al., 2007), and the rate of transcription of *MuRF1* is particularly sensitive to concentric-only exercise (i.e., similar to cycling) in untrained humans at the same (3 h) recovery point (Nedergaard et al., 2007). Considering autophagosome biogenesis is energy-consuming (Plomp et al., 1987) and the UPP favors turnover of short- (i.e., contractile) vs. long-lived (i.e., mitochondrial) proteins, future work should confirm whether UPP-mediated proteolysis is prioritized over autophagy during early recovery from strenuous exercise in humans. Such a temporal response of protein degradation may initially facilitate clearance of cellular debris generated by contractile stress prior to the (mitophagy-dependent) disposal of stressed mitochondria and ultimately, mitochondrial anabolism that peaks ~24 h following exercise (Burd et al., 2012).

Cytoplasmic PINK1was elevated in the control group after 3 h of rest following an overnight fast. This change in PINK1 could indicate that, without the prior mechanical stress of exercise contraction, fasting alone is sufficient to initiate a homeostatic mitophagic response. Indeed, Jamart et al. (2013) previously found that Parkin (PINK1's substrate) was increased in rodents by fasting alone and not after exercise. Another study from the same group reported unchanged PINK1 and Parkin immediately after ultra-endurance exercise in human skeletal muscle (Jamart et al., 2012b). Since mitophagy precedes mitochondrial biogenesis and this mitophagic response is required for efficient oxidative phosphorylation (Qiao et al., 2015; Sin et al., 2016), a brief fast (i.e., ~16 h) may have been sufficient to trigger the removal of mitochondria in preparation for extended periods of energy restriction.

PGC-1α is the principal regulator of mitochondrial biogenesis however its relationship with p53 *in vivo* within the context of exercise adaptation in human skeletal muscle is unknown. For example, PGC-1α has been shown to complex with Tfam in the mitochondria following endurance exercise in rodent skeletal muscle to coordinate mitochondrial biogenesis (Safdar et al., 2011). Furthermore, nuclear export of p53 after exercise is hypothesized to relieve its transcriptional repression of PGC-1α and thereby synergize the synthesis of mtDNA- and nuclear-encoded mitochondrial proteins (Saleem and Hood, 2013). Despite the increased nuclear p53 abundance post-exercise in our study there were no differences in PGC-1α content in the mitochondria, nucleus or cytoplasm following exercise. A study in highly-trained individuals showed nuclear PGC-1α translocation immediately following 90 min continuous cycling (Little et al., 2010). Considering our subjects were untrained and commenced the exercise bout fasted, it is plausible that the 3 h biopsy sampling “window” may have failed to capture some of the earlier, important changes in p53 and PGC-1α activity. A future time course analysis would help to resolve the temporal changes in p53/PGC-1α activity following exercise and the resultant induction of their target genes that are implicated in mitochondrial remodeling.

Limitations of our study methodology should be acknowledged. Firstly, we did not quantify any transcriptional changes in p53 or PGC-1α, and specifically the different PGC-1α isoforms purported to mediate exercise adaptation responses (Ruas et al., 2012). Similarly, due to lack of muscle lysate generated from the fractional extraction process, we were unable to measure the phosphorylation status of several proteins. In this regard, further analysis of p53 phosphorylation responses in the different protein fractions would yield important information to its sub-fraction activation with exercise. Secondly, our findings are limited by our small sample size. While other studies have used similar participant numbers (Little et al., 2010, 2011), this does not preclude the inherent risk of type II error. Studies incorporating greater sample numbers are therefore required to accommodate the innate heterogeneity with Western Blotting using cell fractionation techniques, especially in human skeletal muscle. Thirdly, the extraction of our protein pools was performed on snap frozen tissue. Previous studies, mainly in animals where more muscle tissue can be extracted, have undertaken such analyses on fresh tissue. It is possible the snap freezing and subsequent thaw process may fracture mitochondria and/ or nuclei and therefore selectively limit analysis of these protein pools to those that survived this process. Finally, additional analysis utilizing immunofluorescence microscopy to examine the post-exercise recruitment of Parkin to PINK1 on the mitochondrial membrane may also be warranted to more accurately quantify mitophagy responses rather than conventional measures of total protein abundance.

In conclusion, a single bout of endurance exercise performed following an overnight fast at a moderate intensity (70% VO_2peak_) in untrained individuals' elevated p53 nuclear abundance. This increase in nuclear p53 was presumably a stress response that may promote autophagy although future studies are required to ascertain the biological roles of its specific gene targets under similar experimental conditions. Mitochondrial Atg5 decreased below rest following exercise, suggesting that the UPP may be prioritized over cellular autophagy initially during recovery as a mechanism to remove cellular debris generated by prior contractile stress. This degradative response may precede subsequent mitophagy and ultimately, mitochondrial biogenesis. Indeed, the increased cytoplasmic abundance of PINK1 in the control, fasted group only also suggests that the prolonged withholding of exogenous nutrient availability is sufficient to stimulate a mitophagy-mediated mobilization of endogenous energy. Future studies incorporating a longer time course are required to ascertain the relative contribution from degradative pathways (i.e., proteasomal and lysosomal) following endurance exercise of divergent intensity and determine whether exogenous nutrient availability (i.e., protein or carbohydrate) modulates the prevailing mitochondrial adaptive response.

## Author contributions

JH and SL contributed to the study design. SL conducted clinical trials; BT, DM, and WS performed all data analysis; BT, DM, WS, and JH wrote and reviewed the manuscript. All authors approved the final manuscript.

## Acknowledgements

We thank the study participants for their efforts and dedication. The study was supported by a Collaborative Research Networks (CRN) grant awarded to JH (2013000443).

### Conflict of interest statement

The authors declare that the research was conducted in the absence of any commercial or financial relationships that could be construed as a potential conflict of interest.

## Supplementary material

Supplementary Figure 1 **Stain-free images of total protein loading for mitochondrial, nuclear, and cytoplasmic fractions**.

## References

[B1] Bartlett J. D. Close G. L. Drust B. Morton J. P. (2014). The emerging role of p53 in exercise metabolism. Sports Med. 44, 303–309. 10.1007/s40279-013-0127-9 24264057

[B2] Bartlett J. D. Hwa Joo C. Jeong T. S. Louhelainen J. Cochran A. J. Gibala M. J.. (2012). Matched work high-intensity interval and continuous running induce similar increases in PGC-1alpha mRNA, AMPK, p38, and p53 phosphorylation in human skeletal muscle. J. Appl. Physiol. (1985) 112, 1135–1143. 10.1152/japplphysiol.01040.2011 22267390

[B3] Bartlett J. D. Louhelainen J. Iqbal Z. Cochran A. J. Gibala M. J. Gregson W.. (2013). Reduced carbohydrate availability enhances exercise-induced p53 signaling in human skeletal muscle: implications for mitochondrial biogenesis. Am. J. Physiol. Regul. Integr. Comp. Physiol. 304, R450–R458. 10.1152/ajpregu.00498.2012 23364526

[B4] Burd N. A. Andrews R. J. West D. W. Little J. P. Cochran A. J. Hector A. J.. (2012). Muscle time under tension during resistance exercise stimulates differential muscle protein sub-fractional synthetic responses in men. J. Physiol. 590, 351–362. 10.1113/jphysiol.2011.221200 22106173 PMC3285070

[B5] Camera D. M. Edge J. Short M. J. Hawley J. A. Coffey V. G. (2010). Early time course of Akt phosphorylation after endurance and resistance exercise. Med. Sci. Sports Exerc. 42, 1843–1852. 10.1249/MSS.0b013e3181d964e4 20195183

[B6] Camera D. M. Hawley J. A. Coffey V. G. (2015). Resistance exercise with low glycogen increases p53 phosphorylation and PGC-1alpha mRNA in skeletal muscle. Eur. J. Appl. Physiol. 4, 4 10.1007/s00421-015-3116-x 25650067

[B7] Drake J. C. Wilson R. J. Yan Z. (2016). Molecular mechanisms for mitochondrial adaptation to exercise training in skeletal muscle. FASEB J. 30, 13–22. 10.1096/fj.15-276337 26370848 PMC6137621

[B8] Egan D. F. Shackelford D. B. Mihaylova M. M. Gelino S. Kohnz R. A. Mair W.. (2011). Phosphorylation of ULK1 (hATG1) by AMP-activated protein kinase connects energy sensing to mitophagy. Science 331, 456–461. 10.1126/science.1196371 21205641 PMC3030664

[B9] Feng Z. Hu W. De Stanchina E. Teresky A. K. Jin S. Lowe S.. (2007). The regulation of AMPK beta1, TSC2, and PTEN expression by p53: stress, cell and tissue specificity, and the role of these gene products in modulating the IGF-1-AKT-mTOR pathways. Cancer Res. 67, 3043–3053. 10.1158/0008-5472.CAN-06-4149 17409411

[B10] Fry C. S. Drummond M. J. Glynn E. L. Dickinson J. M. Gundermann D. M. Timmerman K. L.. (2013). Skeletal muscle autophagy and protein breakdown following resistance exercise are similar in younger and older adults. J. Gerontol. A Biol. Sci. Med. Sci. 68, 599–607. 10.1093/gerona/gls209 23089333 PMC3623482

[B11] Gurtler A. Kunz N. Gomolka M. Hornhardt S. Friedl A. A. Mcdonald K.. (2013). Stain-Free technology as a normalization tool in Western blot analysis. Anal. Biochem. 433, 105–111. 10.1016/j.ab.2012.10.010 23085117

[B12] Hawley J. A. Burke L. M. Phillips S. M. Spriet L. L. (2011). Nutritional modulation of training-induced skeletal muscle adaptations. J Appl Physiol (1985) 110, 834–845. 10.1152/japplphysiol.00949.2010 21030665

[B13] Hawley J. A. Hargreaves M. Joyner M. J. Zierath J. R. (2014). Integrative biology of exercise. Cell 159, 738–749. 10.1016/j.cell.2014.10.029 25417152

[B14] Hawley J. A. Zierath J. R. (2004). Integration of metabolic and mitogenic signal transduction in skeletal muscle. Exerc. Sport Sci. Rev. 32, 4–8. 10.1097/00003677-200401000-00002 14748542

[B15] Jamart C. Benoit N. Raymackers J. M. Kim H. J. Kim C. K. Francaux M. (2012a). Autophagy-related and autophagy-regulatory genes are induced in human muscle after ultraendurance exercise. Eur. J. Appl. Physiol. 112, 3173–3177. 10.1007/s00421-011-2287-3 22194006

[B16] Jamart C. Francaux M. Millet G. Y. Deldicque L. Frere D. Feasson L. (2012b). Modulation of autophagy and ubiquitin-proteasome pathways during ultra-endurance running. J Appl Physiol (1985) 112, 1529–1537. 10.1152/japplphysiol.00952.2011 22345427

[B17] Jamart C. Naslain D. Gilson H. Francaux M. (2013). Higher activation of autophagy in skeletal muscle of mice during endurance exercise in the fasted state. Am. J. Physiol. Endocrinol. Metab. 305, E964–E974. 10.1152/ajpendo.00270.2013 23964069

[B18] Kim J. Kundu M. Viollet B. Guan K. L. (2011). AMPK and mTOR regulate autophagy through direct phosphorylation of Ulk1. Nat. Cell Biol. 13, 132–141. 10.1038/ncb2152 21258367 PMC3987946

[B19] Kruiswijk F. Labuschagne C. F. Vousden K. H. (2015). p53 in survival, death and metabolic health: a lifeguard with a licence to kill. Nat. Rev. Mol. Cell Biol. 16, 393–405. 10.1038/nrm4007 26122615

[B20] Lira V. A. Okutsu M. Zhang M. Greene N. P. Laker R. C. Breen D. S.. (2013). Autophagy is required for exercise training-induced skeletal muscle adaptation and improvement of physical performance. FASEB J. 27, 4184–4193. 10.1096/fj.13-228486 23825228 PMC4046188

[B21] Little J. P. Safdar A. Bishop D. Tarnopolsky M. A. Gibala M. J. (2011). An acute bout of high-intensity interval training increases the nuclear abundance of PGC-1alpha and activates mitochondrial biogenesis in human skeletal muscle. Am. J. Physiol. Regul. Integr. Comp. Physiol. 300, R1303–R1310. 10.1152/ajpregu.00538.2010 21451146

[B22] Little J. P. Safdar A. Cermak N. Tarnopolsky M. A. Gibala M. J. (2010). Acute endurance exercise increases the nuclear abundance of PGC-1alpha in trained human skeletal muscle. Am. J. Physiol. Regul. Integr. Comp. Physiol. 298, R912–R917. 10.1152/ajpregu.00409.2009 20106991

[B23] Louis E. Raue U. Yang Y. Jemiolo B. Trappe S. (2007). Time course of proteolytic, cytokine, and myostatin gene expression after acute exercise in human skeletal muscle. J Appl Physiol (1985) 103, 1744–1751. 10.1152/japplphysiol.00679.2007 17823296

[B24] Maiuri M. C. Galluzzi L. Morselli E. Kepp O. Malik S. A. Kroemer G. (2010). Autophagy regulation by p53. Curr. Opin. Cell Biol. 22, 181–185. 10.1016/j.ceb.2009.12.001 20044243

[B25] Matoba S. Kang J. G. Patino W. D. Wragg A. Boehm M. Gavrilova O.. (2006). p53 regulates mitochondrial respiration. Science 312, 1650–1653. 10.1126/science.1126863 16728594

[B26] Mizushima N. Klionsky D. J. (2007). Protein turnover via autophagy: implications for metabolism. Annu. Rev. Nutr. 27, 19–40. 10.1146/annurev.nutr.27.061406.093749 17311494

[B27] Mizushima N. Yoshimori T. Ohsumi Y. (2011). The role of Atg proteins in autophagosome formation. Annu. Rev. Cell Dev. Biol. 27, 107–132. 10.1146/annurev-cellbio-092910-154005 21801009

[B28] Narendra D. P. Jin S. M. Tanaka A. Suen D. F. Gautier C. A. Shen J.. (2010). PINK1 is selectively stabilized on impaired mitochondria to activate Parkin. PLoS Biol. 8:e1000298. 10.1371/journal.pbio.1000298 20126261 PMC2811155

[B29] Nedergaard A. Vissing K. Overgaard K. Kjaer M. Schjerling P. (2007). Expression patterns of atrogenic and ubiquitin proteasome component genes with exercise: effect of different loading patterns and repeated exercise bouts. J. Appl. Physiol. (1985) 103, 1513–1522. 10.1152/japplphysiol.01445.2006 17690190

[B30] Park J. Y. Wang P. Y. Matsumoto T. Sung H. J. Ma W. Choi J. W.. (2009). p53 improves aerobic exercise capacity and augments skeletal muscle mitochondrial DNA content. Circ. Res. 105, 705–712. 10.1161/CIRCRESAHA.109.205310 19696408 PMC2761626

[B31] Perez-Schindler J. Philp A. (2015). Regulation of skeletal muscle mitochondrial function by nuclear receptors: implications for health and disease. Clin. Sci. 129, 589–599. 10.1042/CS20150246 26186742

[B32] Plomp P. J. Wolvetang E. J. Groen A. K. Meijer A. J. Gordon P. B. Seglen P. O. (1987). Energy dependence of autophagic protein degradation in isolated rat hepatocytes. Eur. J. Biochem. 164, 197–203. 10.1111/j.1432-1033.1987.tb11011.x 3830181

[B33] Qiao S. Dennis M. Song X. Vadysirisack D. D. Salunke D. Nash Z.. (2015). A REDD1/TXNIP pro-oxidant complex regulates ATG4B activity to control stress-induced autophagy and sustain exercise capacity. Nat. Commun. 6, 7014. 10.1038/ncomms8014 25916556 PMC4421852

[B34] Ruas J. L. White J. P. Rao R. R. Kleiner S. Brannan K. T. Harrison B. C.. (2012). A PGC-1alpha isoform induced by resistance training regulates skeletal muscle hypertrophy. Cell 151, 1319–1331. 10.1016/j.cell.2012.10.050 23217713 PMC3520615

[B35] Safdar A. Little J. P. Stokl A. J. Hettinga B. P. Akhtar M. Tarnopolsky M. A. (2011). Exercise increases mitochondrial PGC-1alpha content and promotes nuclear-mitochondrial cross-talk to coordinate mitochondrial biogenesis. J. Biol. Chem. 286, 10605–10617. 10.1074/jbc.M110.211466 21245132 PMC3060512

[B36] Saleem A. Adhihetty P. J. Hood D. A. (2009). Role of p53 in mitochondrial biogenesis and apoptosis in skeletal muscle. Physiol. Genomics 37, 58–66. 10.1152/physiolgenomics.90346.2008 19106183

[B37] Saleem A. Hood D. A. (2013). Acute exercise induces tumour suppressor protein p53 translocation to the mitochondria and promotes a p53-Tfam-mitochondrial DNA complex in skeletal muscle. J. Physiol. 591, 3625–3636. 10.1113/jphysiol.2013.252791 23690562 PMC3731618

[B38] Schwalm C. Jamart C. Benoit N. Naslain D. Premont C. Prevet J.. (2015). Activation of autophagy in human skeletal muscle is dependent on exercise intensity and AMPK activation. FASEB J. 29, 3515–3526. 10.1096/fj.14-267187 25957282

[B39] Sin J. Andres A. M. Taylor D. J. Weston T. Hiraumi Y. Stotland A.. (2016). Mitophagy is required for mitochondrial biogenesis and myogenic differentiation of C2C12 myoblasts. Autophagy 12, 369–380. 10.1080/15548627.2015.1115172 26566717 PMC4836019

[B40] Singh R. Cuervo A. M. (2011). Autophagy in the cellular energetic balance. Cell Metab. 13, 495–504. 10.1016/j.cmet.2011.04.004 21531332 PMC3099265

[B41] Smiles W. J. Areta J. L. Coffey V. G. Phillips S. M. Moore D. R. Stellingwerff T.. (2015). Modulation of autophagy signaling with resistance exercise and protein ingestion following short-term energy deficit. Am. J. Physiol. Regul. Integr. Comp. Physiol. 309, R603–R612. 10.1152/ajpregu.00413.2014 26136534

[B42] Tanida I. Ueno T. Kominami E. (2008). LC3 and Autophagy. Methods Mol. Biol. 445, 77–88. 10.1007/978-1-59745-157-4_4 18425443

[B43] Twig G. Elorza A. Molina A. J. Mohamed H. Wikstrom J. D. Walzer G.. (2008). Fission and selective fusion govern mitochondrial segregation and elimination by autophagy. EMBO J. 27, 433–446. 10.1038/sj.emboj.7601963 18200046 PMC2234339

[B44] Vives-Bauza C. Zhou C. Huang Y. Cui M. De Vries R. L. Kim J.. (2010). PINK1-dependent recruitment of Parkin to mitochondria in mitophagy. Proc. Natl. Acad. Sci. U.S.A. 107, 378–383. 10.1073/pnas.0911187107 19966284 PMC2806779

[B45] Yan Z. Lira V. A. Greene N. P. (2012). Exercise training-induced regulation of mitochondrial quality. Exerc. Sport Sci. Rev. 40, 159–164. 10.1097/JES.0b013e3182575599 22732425 PMC3384482

