# Supplementary figures and images for: Acute Endurance Exercise Induces Nuclear p53 Abundance in Human Skeletal Muscle
Source: Front Physiol. 2016 Apr 26;7:144. doi: 10.3389/fphys.2016.00144 (PMC4845512; doi:10.3389/fphys.2016.00144)

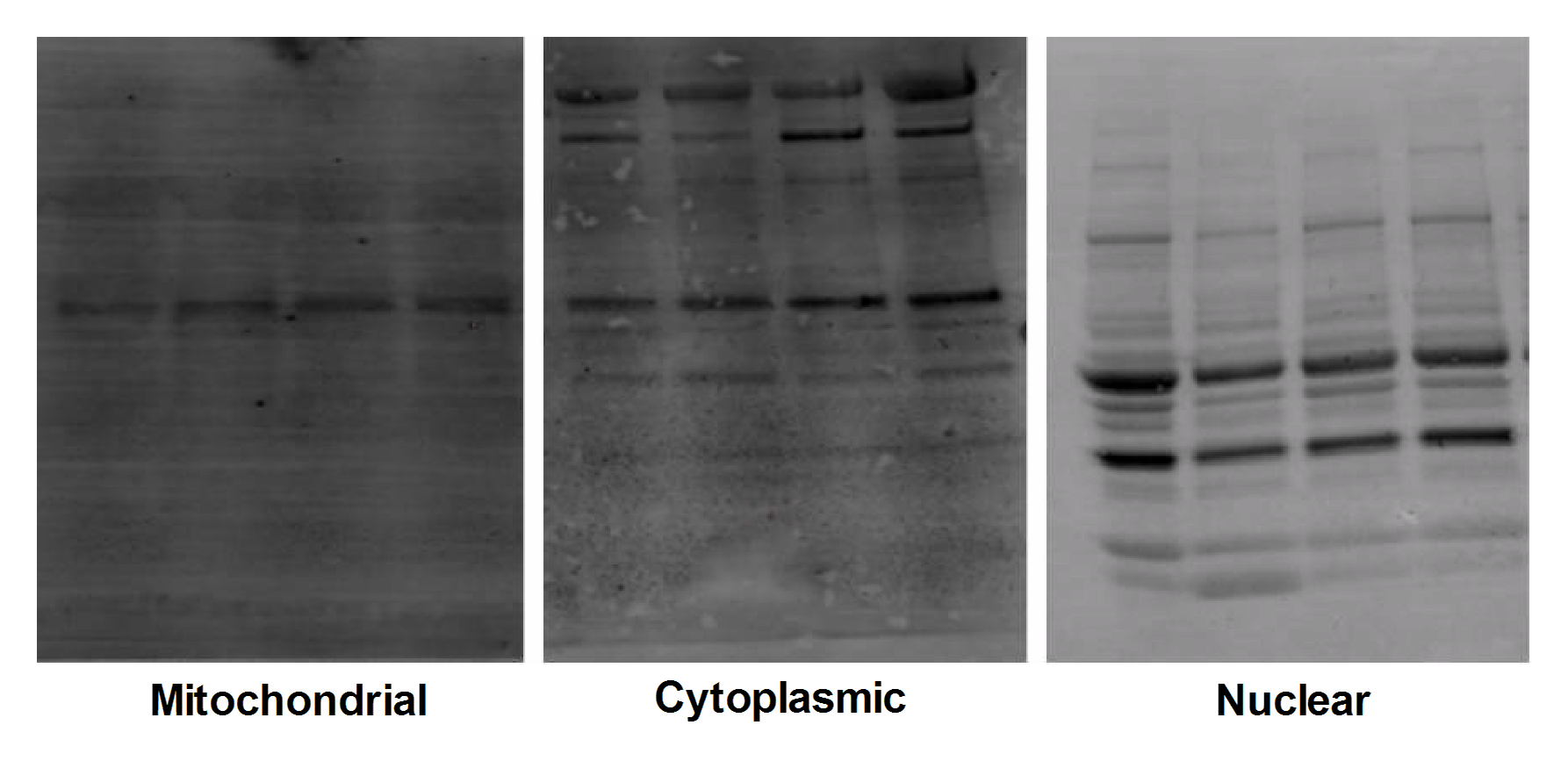

Supplement: Supplementary Figure 1 — Stain-free images of total protein loading for mitochondrial, nuclear, and cytoplasmic fractions. [file Image1.TIF]
